# In Vitro Investigation of the Effects of the Food Additives Monosodium Glutamate and Allura Red AC on the human Gut Microbiota and Intestinal Cell Lines

**DOI:** 10.1111/1750-3841.70504

**Published:** 2025-08-24

**Authors:** Adela Granja‐Iglesias, Xenia Vázquez, Carlos Sabater, Arancha Hevia, Manuel Garrido‐Romero, Ana Muñoz‐Labrador, Plácido Galindo‐Iranzo, Rosa Lebrón‐Aguilar, Jesús E. Quintanilla‐López, F. Javier Moreno, Lorena Ruiz, Patricia Ruas‐Madiedo

**Affiliations:** ^1^ Group of Functionality and Ecology of Beneficial Microorganisms (MicroHealth) Dairy Research Institute of Asturias (IPLA‐CSIC) Oviedo Asturias Spain; ^2^ Health Research Institute of Asturias (ISPA) Avenida Hospital Universitario s/n Oviedo Asturias Spain; ^3^ Group of Chemistry and Functionality of Carbohydrates and Derivatives, Institute of Food Science Research, CIAL (CSIC‐UAM), Nicolás Cabrera, 9, Campus de Cantoblanco Universidad Autónoma de Madrid Madrid Spain; ^4^ Group of Photolysis and Chromatography Institute of Physical Chemistry ‘Blas Cabrera’ (IQF‐CSIC) Madrid Spain

**Keywords:** AR, food additives, microbiota, MSG

## Abstract

This work addresses in vitro the potential impact of two food additives in the safety spotlight, i.e., the flavor enhancer monosodium glutamate (MSG) and the azo‐dye colorant allura red AC (AR), on the human gut microbiota from healthy donors by using *in* *vitro* faecal batch culture models, and an intestinal cell line model. The rationale to investigate MSG and AR safety on the gut microbiota is motivated by recent research pointing to a contribution of their chronic consumption to intestinal inflammation. The short‐term exposure of faecal samples to the individual additives at two single doses (0.8 and 0.08 mM for MSG; and 0.6 and 0.06 mM for AR) did not exert significant changes in the relative abundance of gut bacteria as determined through 16S rRNA sequencing. MSG and AR were degraded into γ‐aminobutyric acid (GABA) and 4‐amino‐5‐methoxy‐2‐methylbenzenesulfonic acid (4A5M2M), respectively, during fermentation. While GABA was exclusively derived from microbial activity, a partial degradation of AR to 4A5M2M was triggered by heat treatment of AR‐containing samples prior to faecal fermentation. Further, MSG and AR alone or after exposure to faecal samples did not show cytotoxic effects on intestinal HT29 cell monolayers. These results highlight the role of the human gut bacteria and the impact of heat‐based processing in the bioconversion of certain food additives. Our findings reinforce the safety of both food additives regarding their impact on the gut microbial communities under conditions resembling acute short‐term exposures, although the potential biological effects of the metabolites generated from their degradation or from chronic exposures should be further investigated.

## 1 Introduction

Diet intimately shapes our gut microbiota, consequently affecting its impact on human health beyond the intestinal level, influencing metabolism, immunity, and several extra‐intestinal functions. Accordingly, to date, diet has been recognized as one of the most influential modulators of the human gut microbiome (Bourdeau‐Julien et al. 2023), and dietary‐based interventions are the approach most widely investigated to rebalance the gut microbiome in diverse conditions (Armet et al. 2021; Cunningham et al. 2021). Nonetheless, classical food safety risk assessment approaches have dismissed the possible role that non‐absorbable compounds may exert on human health, considering they are inert following excretion. This scenario ignores the contribution of the gut microbiome to the metabolization of non‐absorbable dietary compounds, and the potential impact of this microbial biotransformation on human health (Garrido‐Romero et al. 2024).

Notably, investigations arisen in recent years have begun to explore the potential effects that certain food additives and chemical compounds ingested, voluntarily or involuntarily, through food may have on the human gut microbiome (Jimenez Loayza et al. 2023; Gerasimidis et al. 2020; Roca‐Saavedra et al. 2018). As a matter of fact, some of these investigations have demonstrated that certain chemicals acquired through diet can affect the gut microbiome ecosystem, sometimes being even bioconverted by the gut microbiota, although the possible implications of these effects on human health are not fully clear. For instance, some stabilizers and artificial sweeteners can affect beneficial bacterial taxa inducing, glucose intolerance, expansion of taxa associated with recurrence in Crohn's disease or reduction of the microbiome fiber fermentation capacity (Jimenez Loayza et al. 2023; Gerasimidis et al. 2020; Roca‐Saavedra et al. 2018). Although some of the effects were dependent on the basal individual microbiome configuration, the detrimental impact of some food additives on the microbiome has been confirmed through in vitro investigations (Chassaing et al. 2022). Food additives that have shown effects on the gut microbiome in various investigations include the flavor enhancer MSG, E621, and the azo‐dye colorant AR, E129. Both food additives are widely used in processed foods commonly consumed in Westernized populations diets, however available results are not consistent across investigations. In light of the critical influence of the diet‐microbiome‐health interplay, further investigation is warranted so as to ensure their safety for the gut microbiome and human health, motivating us to conduct this investigation on these two specific food additives: MSG and AR.

MSG is a common flavor enhancer, structurally derived from L‐glutamic acid, a naturally occurring amino acid. EFSA has established a safe, acceptable daily intake (ADI) for MSG of 30 mg/kg bw per day (EFSA 2017). Noticeably, controversy exists regarding the potential detrimental effects of MSG on the intestinal microbiome. While some studies have demonstrated no relevant effects of recommended consumption doses on the microbiome in various *in* *vitro* and animal models (Jinzhao et al. 2022) or in human intervention trials (Peng et al. 2018), other investigations have shown potential adverse effects on human health that may be microbiota‐mediated. For instance, MSG consumption at high doses or in combination with high‐fructose and high‐fat diets has demonstrated detrimental effects on the microbiome and capacity to exacerbate dysbiosis and disease risk in male hamsters (Pongking et al. 2020). MSG consumption in male rats has also been associated with microbiome shifts together with hepatic and renal metabolic changes (e.g., increased trimethylamine in association to kidney injury) (Nahok et al. 2021). However, potential health benefits for MSG consumption have also been proposed since this compound is a precursor for microbial production of γ‐aminobutyric acid (GABA), a neurotransmitter whose deficiency has been associated to various brain alterations (Duranti et al. 2020), and that could further support an alternative via for modulation of the microbial populations in the gut ecosystem (Strandwitz et al. 2019).

In relation to AR, it is an azo‐dye synthetic colorant whose chronic consumption has been associated with exacerbation in experimental models of induced colitis, through both microbiota‐dependent and independent pathways (Kwon et al. 2022). Some models have postulated that the effect is linked to the dye metabolization through azo‐reductases encoded in certain gut bacterial species into two major metabolites (He et al. 2021), although their contribution to disease risk upon consumption in humans remains unclear. For the colorant AR, the ADI recommended by EFSA for any population is 7 mg/kg bw per day (EFSA Panel ANS 2009; EFSA Panel ANS 2015; EFSA Panel ANS 2017).

This investigation sustains on previous research pointing to limited or null digestion and absorption of MSG and AR upon ingestion, resulting in a potential impact on the gut microbiota. In the particular case of AR, negligible amounts may be absorbed and most of the additive is excreted with feces in the form of different metabolites, therefore supporting most of the ingested AR reach the colon (Ramesh and Muthuraman 2018; Joint FAO/WHO Expert Committe on Food Additives, 2016) where it can potentially interact with the intestinal microbiota. Similarly, studies with mice have evidenced that ingested MSG may partially reach the colon, potentially interacting with the microbiota at this location (Xu et al. 2022). Indeed, the impact of MSG and AR on the gut microbiota has recently started to be investigated, although available studies are limited and mostly restricted to mice models (Kwon et al. 2022; Xu et al. 2022). Despite the advantages of *in* *vivo* models as compared to *in* *vitro* models like the ones herein employed, mice models have demonstrated noticeable differences in their gut microbiomes, hampering direct translation of results from such models to those observed in the human gut microbiota (Zhou et al. 2023; Nguyen et al. 2015), and hence studies with human microbiota and human cell models may be useful to further investigate this matter.

It is important to consider that flavoring, colorant, preservative, or antioxidant agents are integral components of the manufacturing process to enhance food quality and perception. These additives are typically incorporated at various stages of production, including processing, preservation treatments, packaging, storage, as well as the cooking unit process required for the final consumption (Ramesh and Muthuraman 2018). Food additives like AR can be found in a wide range of foods, such as alcoholic and non‐alcoholic products, confectionery, meat products, sauces, cheeses, and so forth (Rovina et al. 2016; Mortensen et al. 2017). And hence may be subjected to various thermal or technological treatments both at industrial processing and/or during cooking in household settings which could potentially affect food additives structure and/or their effects upon consumption. However, the consideration of the effect of processing on AR has been neglected up to date.

In this work, we have taken advantage of an experimental design using an *in* *vitro* faecal batch culture model, in combination with targeted metabolomics, as well as an intestinal cell line model, to further investigate the potential impact of previously autoclaved MSG and AR on the human gut microbiota from self‐reported healthy donors and consequences for the gut ecosystem wellbeing.

## 2 Material and Methods

### 2.1 Ethics Statement and Faecal Samples Collection

Faecal samples were provided by six healthy donors (three males and three females) aged between 24 and 37 years who reported no known chronic, infectious diseases or medication at the time of the sample collection. All donors were informed about the experiment's objective and provided written informed consent. Sample collection was approved by the Research Ethics Committee of the Principality of Asturias (authorization number: CEImPA 2022.441).

### 2.2 *in* *vitro* Batch Faecal Cultures

Faecal samples were processed within 1 h from deposition. Each faecal sample was incubated in a basal fermentation medium (BFM). This medium contained (2 g/L) peptone water, (2 g/L) yeast extract, (0.1 g/L) NaCl, (0.04 g/L) K_2_HPO_4_, (0.04 g/L) KH_2_PO_4_, (0.01 g/L) MgSO_4_·7H_2_O, (0.01 g/L) CaCl_2_·2H_2_O, (0.01 g/L) NaHCO_3_, (0.5 g/L) L‐cysteine‐HCl monohydrate, (0.5 g/L) bile salts, (2 mL/L) tween 80, (0.2 g/L) glucose, (0.5 g/L) fructose, (0.5 g/L) cellobiose, and (0.5 g/L) maltose. It was adjusted to pH 6.8 before autoclave sterilization (121°C). After sterilization, (100 µL/L) vitamin K and (50 mg/L) hemin were added from filtered‐sterilized stocks. The additives MSG (specifically, L‐glutamic acid monosodium salt monohydrate, molecular weight 187.13 g/mol) and AR (disodium 6‐hydroxy‐5‐[(2‐methoxy‐5‐methyl‐4‐sulfophenyl)azo]‐2‐naphthalenesulfonate; molecular weight 496.42 g/mol), with an 80% purity, were from Sigma‐Merck (Darmstadt, Germany). In order to ensure the sterility of the faecal fermentation medium and avoid incorporating any undesirable contaminant with the additives, they were added to the BFM to be autoclaved for the subsequent *in* *vitro* fermentation. The AR‐derived metabolites 4‐amino‐5‐methoxy‐2‐methylbenzenesulfonic acid (4A5M2M) and 1‐amino‐2‐naphthol‐6‐sulfonic acid (1A2N6S) were obtained from Biosynth (Compton, United Kingdom). The rest of the used reagents were from Sigma‐Merck.

For the experimental design, each faecal sample was incubated in parallel under five different conditions: BFM free from additives (control sample), BFM supplemented with two different doses of MSG and BFM supplemented with two different doses of AR. The doses of additives employed were selected taking into consideration the recommended ADI for each of the additives by EFSA, and assuming a donor standard weight of 75 kg and 150 g stool deposition per day. In the case of MSG, a daily ingestion equivalent to the established ADI (30 mg/kg bw/day) and a donor standard weight of 75 kg, gives 2.25 g of MSG of daily dose; if it is assumed that no MSG absorption would take place at intestinal level, then the same amount of MSG would be excreted daily in 150 g faeces (15 mg MSG/ g faeces = dose 1 = MSG‐ADI). However, since it is known that glutamate is absorbed along the gastrointestinal tract and that only a percentage of ingested MSG will reach the colon, we selected a second dose ten times lower than ADI (dose 2 = MSG‐low). Therefore, considering that in our experimental design 1 g of faeces was homogenized in 10 mL of a PBS solution and this will be diluted ten times after the BFM inoculation, the final concentration tested was 0.15 mg MSG/mL (0.8 mM) for dose 1 and 0.015 mg MSG/mL (0.08 mM) for dose 2, which were added to the BFM from a 100x‐stock solution (15 mg/mL) of MSG in ultrapure water. Following the same rationale, EFSA accepts a daily intake of 7 mg/kg bw/day of AR; then, for a donor of 75 kg weight, the maximum AR recommended is 0.525 g and assuming no AR absorption the excretion in 150 g faeces of AR will be 3.5 mg/g (= dose 1 = AR‐ADI). Azo dyes are artificial compounds that may not be absorbed, so they presumably arrive at the colon. Hence, an over‐dose equivalent to ten times higher than the ADI (dose 2 = AR‐high) was used. A 1000x‐stock solution (35 mg/mL) of AR in ultrapure water was prepared and the final concentrations tested in the BFM inoculated with the diluted faeces were 0.06 mM (dose 1) and 0.6 mM (dose 2).

The solution for faecal homogenization, BFM and food additives were pre‐reduced for 24 h prior to the sample collection and processing inside an MG500 anaerobic chamber (Don Whitley Scientific, West Yorkshire 100, UK), with an atmosphere of 10% (v/v) H_2_, 10% CO_2_, and 80% N_2_). Faeces were homogenized (1:10 w/v) in pre‐reduced PBS + 0.25% L‐cysteine. Then, 8 mL of each homogenate was used to inoculate 80 mL of pre‐reduced media (BFM, or BFM supplemented with the above‐described doses of MSG or AR) to have a total of 5 culture vessels per donor. Cultures were performed at 37°C in the anaerobic chamber under constant mild agitation (125 rpm) for 24 h.

### 2.3 Samples Collection From Faecal Fermentations and Processing for Microbiota Analyses

Culture vessels were sampled 5 min after inoculation of the homogenized faecal samples in the corresponding BFM media, and after 24 h of incubation under anaerobic conditions as above described. Samples collected were centrifuged at 11,500 rpm for 5 min to obtain supernatants and pellets, the latter washed twice with PBS. Both were kept properly frozen at –20°C. After 24 h of incubation, the control vessel (BFM without additives) and those incubated in the presence of additives (indicated above) were collected to repeat the same procedure. Pellets were used for DNA isolation and sequencing of the 16S rRNA gene, whereas the supernatants were used to quantify short‐chain fatty acids (SCFA) and metabolites derived from the microbial metabolization of the food additives under investigation, as well as for toxicity experiments in an intestinal cell line model. A total of 36 samples, for both supernatants and pellets, were collected.

#### 2.3.1 Viable Counts and pH Determination

GAM culture medium (Nissui Pharmaceutical CO., LTD. Japan) with 1.5% agar was used for performing microbiological counts of viable and culturable total bacteria along the faecal cultivation. The pH was measured on faecal supernatants.

#### 2.3.2 DNA Extraction and Sequencing

Partial 16S rRNA analysis was performed on 36 samples of six different faecal donors in six different conditions per donor: 1 basal (0 h) sample and 5 (different additives and doses) final (24 h) samples. To this end, DNA was extracted from pellets collected from the cultures using the Power Soil ProKit (Qiagen GmbH, Hilden, Germany), with some modifications as previously described (Calvete‐Torre et al. 2022). Briefly, cell pellets collected from faecal fermentation were dissolved with 750 µL of a lysis solution and incubated at 65°C for 10 min. Afterwards, samples were mechanically lysed through bead‐beating using beads‐containing tubes and a vortex adapter (Ref 13000‐V1‐24, Qiagen GmbH) for lysis. The rest of the DNA isolation protocol was performed according to the manufacturer's instructions. Primers 16S‐ProV3V4‐forward (CCTACGGGNBGCASCAG) and 16S‐ProV3V4‐reverse (GACTACNVGGGTATCTAATCC) were used for V3‐V4 region sequencing in an Illumina MiSeq instrument, in the Sequencing Facilities of “Instituto de Parasitología y Biomedicina López Neyra” (Granada, Spain) as previously described (Calvete‐Torre et al. 2022), achieving a sequencing depth of at least 10 million reads per sample.

#### 2.3.3 Bioinformatic and Statistical Analysis

In this study, 16S rRNA reads matched by pair‐ends were quality filtered and classified into amplicon sequence variants (ASVs) using QIIME2 software v2021.8 to study the micro biota composition of samples (Bolyen et al. 2019). Quality control filtering was performed, keeping sequences with a mean sequence quality score >20. Taxonomy classification was performed using the reference database SILVA 138 release, and all reads were classified to the genus level. Microbiome R package (Lahti and Shetty 2017) was used for alpha diversity estimators’ (Chao1, Shannon, Simpson and Inverse Simpson) calculations and to perform general statistical analysis of microbiota composition. An additional beta‐diversity analysis of microbial communities was performed following the Bray‐Curtis dissimilarity method (Lozupone and Knight 2005), implemented in Phyloseq R package (McMurdie and Holmes 2013). Bray‐Curtis dissimilarity was also used to cluster microbiota composition profiles of samples under study. Ordination plots describing the distribution of microbial communities across individuals were generated using Microbiome R package (Lin and Peddada 2020). Statistic differences in microbial composition at different fermentation times (0 and 24 h) and conditions (control, MSG‐ADI corresponding to 0.8 mM; MSG‐low corresponding to 0.08 mM; AR‐ADI corresponding to 0.06 mM; AR‐high corresponding to 0.6 mM) were calculated using microbiome‐specific statistical methods ANCOM and LEfSE methods and MicrobiomeMarker R package (Lin and Peddada 2020; Cao et al. 2022). For this purpose, statistically significant (*p* < 0.05 and *padj* < 0.25 corrected by Holm method) differences in microbiota composition (i.e., normalized microbial read counts) at initial (0 h) and final (24 h) fermentation times were calculated for each substrate. It should be noted that sequencing reads were normalized prior to differential analysis using total sum scaling (TSS) method implemented in microbiomeMarker R package.

To further investigate the effect of interindividual variability in microbiota composition profiles, linear mixed‐effects models (LMMs) were computed using lme4 R package. Specifically, LMM models were computed to assess statistical differences (*p* < 0.05 adjusted by Holm method) in the beta‐diversity values (expressed as Bray‐Curtis dissimilarity metric) and microbial genera abundance (expressed as percentages) of different groups of samples (Basal, Control, AR‐ADI, AR‐high, MSG‐ADI, and MSG‐low). Experimental groups and substrate doses were considered as fixed effects while donor and sex were considered as random effects. Post‐Hoc analysis of LMM model (Tukey test) was performed using multcomp R package.

Potential associations between bacterial genera showing an increment in their abundances after faecal fermentation were determined using ccrepe and qgraph R packages (Epskamp et al. 2012; Schwager et al. 2019). Finally, Pearson correlation coefficients between microbial genera showing significant (*p* < 0.05) increments in their abundance after fermentation were calculated. All statistical tests and models were performed on R (v4.1.1) or IBM SPSS Statistic + for Window version 28.0 (IBM Corp., NY, USA). Reads generated in this investigation have been deposited in the short reads archives of the NCBI, under accession number PRJNA1178457.

#### 2.3.4 Short Chain Fatty Acids Analysis by GC/FID

Analysis of SCFA was performed as previously described (Calvete‐Torre et al. 2022). In short, cell free‐supernatants (100 µL) from the 36 samples were mixed with 50 µL 20% v/v formic acid, 450 µL methanol and 50 µL internal standard solution (2‐ethylbutyric acid, 80 µg/mL). Faecal supernatants obtained after centrifugation were used for SCFA quantification (mM) in an Agilent 6890 gas chromatograph with an automatic injection module, a flame ionization detector (FID) and the ChemStation Agilent software (Agilent Technologies, CA, USA). Separation was performed on an HP‐FFAP (30 m × 0.25 mm × 0.25 µm) column (Agilent) using He as carrier gas at a constant flow rate of 1.3 mL/min, using temperature ramps ranging from 100°C to 170°C (6°C/min), and then ramping up to 240°C (25°C/min). Samples (1 µL) were injected in split mode (1:20 ratio) at 240°C.

### 2.4 Determination of Additives and Metabolites Resulting From Their Microbial Metabolization by LC

#### 2.4.1 Determination of MSG and GABA by HPLC/PDA

MSG and its microbial metabolite GABA were quantified by HPLC, as previously described (Duranti et al. 2020), using filtered faecal supernatants after derivatization with diethyl ethoxymethylenemalonate (DEEM, Sigma‐Merck). Both metabolites were quantified using a chromatographic system composed of the Alliance 2695 separation module, the UV‐visible PDA 2996 detector (set at 280 nm) and the acquisition/ analysis software Empower (Waters, Milford, MA, USA). Separation was carried out in the Ascentis C18 (250 × 4.6 mm, 5 µm) reverse‐phase column with a pre‐column Supelguard Ascentis C18 (20 × 4.0 mm) (Supelco, Sigma‐Aldrich, MO, USA), at 35°C and 1 mL/min flow rate. The mobile phase consisted in a gradient of 25 mM acetate buffer (pH 6.7, with 0.02% sodium azide; eluent A), acetonitrile (eluent B) and methanol (eluent C). Quantification (mM) was performed using calibration curves (linearity coefficient of correlation R^2^ >0.99), made with different concentrations of MSG or GABA standards (Sigma) submitted to the same derivatization procedure.

#### 2.4.2 Determination of AR and Derived Metabolites by LC‐MS

An LC‐MS method for determination of the food additive AR, and its derived metabolites 4A5M2M and 1A2N6S was optimized using an Agilent 1100 Series LC system (Agilent Technologies) with an auto sampler, a quaternary pump and a column oven coupled to an HTC‐Ultra ETD II ion trap mass spectrometer (Bruker Daltonics, Fremont, USA) with an electrospray (ESI) interface. The Bruker Compass 1.2 software (Bruker Daltonics) was used for data acquisition and processing.

The separation was performed on a C18 HyPURITY column (100 × 2.1 mm, 3 µm particle size; Thermo Fischer Scientific, Waltham, USA) with a C18 HyPURITY pre‐column (10 × 2.1 mm, 3 µm; Thermo Fischer Scientific) at 25°C and at a flow rate of 0.2 mL/min. Water (Millipore, Billerica, USA; eluent A), methanol (OPTIMA LC/MS grade, Thermo Fisher Scientific; eluent B) and a 6.8 pH buffer of ammonium acetate 129 mM (Sigma‐Merck; eluent C) were used with the following optimized gradient: 4 min in isocratic conditions (90% A, 5% B, 5% C), then the percentage of B was linearly increased up to 95% in 10 min (decreasing the percentage of A accordingly and maintaining constant the 5% of C), kept for 15 min and then returned to the original proportion within 1 min.

The ESI source parameters were optimized as follows: spray voltage was set at 2.1 kV, nebulizer gas at 40 psi, and drying gas at 12 L/min and 350°C. Nitrogen (99.5% purity) was used as nebulizer and drying gas, while helium (99.999%) was used as the collision gas in the MS/MS experiments. Full scan mass spectra from m/z 150 to 700 were recorded working in the negative ion mode. MS/MS spectra were obtained by collision induced dissociation (CID) of the corresponding [M‐H] ¯ precursor ions, applying fragmentation amplitudes between 0.9 and 1.1.

Identity of AR and their metabolites in the samples was confirmed by matching their retention times and their MS and MS/MS spectra with those obtained for the standards. Quantification (mM) was performed on the extracted full scan [M‐H] ¯ traces, using calibration curves based on matrix‐matched standards (BFM fortified with AR or 4A5M2M) in the 1–10 mg/L range. 5 µL of the calibration solutions and the faecal samples were injected into the LC‐MS system, diluting the sample when necessary prior to injection.

### 2.5 Impact of the Food Additives and Microbial Derivatives on Host

#### 2.5.1 Cell Line and Culture Conditions

Intestinal epithelial cell line HT29 (ECACC No. 91072201, European Collection of Cell Cultures, Salisbury, UK) was grown in McCoy's medium supplemented with 10% (v/v) heat‐inactivated bovine fetal serum and a mixture of antimicrobials (50 µg/mL penicillin, 50 µg/mL streptomycin, 50 µg/mL gentamicin, and 1.25 µg/mL amphotericin B). Standard sub‐culturing and maintenance of the cell line was performed, according to procedures previously described (Valdés et al. 2015). For this work, HT29 was used in the passage 149 and 150 in order to perform two replicated experiments.

#### 2.5.2 HT29 Cytotoxicity by RTCA‐DP

HT29 cells behavior upon exposure to cell‐free faecal supernatants from the 36 samples was assessed by using a real‐time cell analyzer (RTCA‐DP) xCelligence equipment (ACEA Bioscience, Agilent Technologies, CA, USA), as previously reported (Schwager et al. 2019). In short, E‐plates (ACEA Bioscience, Agilent Technologies) were seeded with 2 × 10^5^ HT29 cells per well (in 100 µL of culture media) and held in the RTCA device, previously placed in an incubator (at 37°C with 5% CO2). The impedance (or cell index), which measures the attachment/detachment and morphological changes in the cells, was monitored for about 20 h to follow the formation of the monolayer until a confluent state. Then, medium was removed from each well and fresh McCoy´s containing 40% filtered faecal supernatants was added. In addition to the 36 samples, several control media were used: McCoy´s (MM, used as reference), BFM without additives, BFM with the highest concentration of MSG, and BFM with highest concentration of the AR. The impedance monitoring was recorded every 10 min for an additional 24 h under the same incubation conditions. Finally, data of cell index were normalized to a baseline (value “0”), using the reference values of MM control and the first measurement point after the addition of samples or controls.

## 3 Results

### 3.1 Microbiota Analysis in Faecal Cultures

Microbial growth in faecal cultures was first monitored through colony count determination of viable cells and pH measurement, demonstrating significant interindividual variability in the fermentation dynamics, influenced by the faecal donor (Supplementary Figure S1). However, no significant differences were detected in any of these two parameters when comparing the cultures with the different food additives/doses, with the control culture.

**FIGURE 1:**
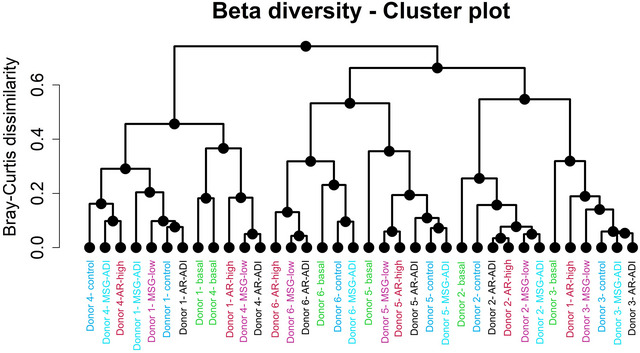
Clustering analysis of taxonomic profiles found in the microbiota belonging to donors in the presence and/or absence of MSG and AR. The Bray‐Curtis dissimilarity method was selected for the calculation. As can be seen, few samples corresponding to the same group were clustered together. These results highlight the role of interindividual variability.

The 16S rRNA‐sequencing allowed discerning microbiota profiling along the faecal fermentations at genus level. Alpha diversity estimators, including Chao1, Shannon, Simpson, and inverse Simpson indices, were calculated to measure the variability of species within samples in basal conditions (time 0 h; Supplementary Figure S2). These indices reflected different interindividual patterns in the microbiota diversity in different donors. In this regard, similar alpha diversity metrics were observed for 2, 5, and 6. Then, beta diversity estimators based on Bray–Curtis distances were calculated to account for differences between individuals and treatment groups. Beta diversity values calculated for taxonomic composition data were similar in all treatment groups (Supplementary Figure S3), not showing significant differences (*p* > 0.05). In addition, sample clustering based on Bray‐Curtis distances demonstrated cluster groups strongly dependent on sample donors (Figure 1), with some exceptions. In this regard, most samples from the same donor were clustered together, although some samples corresponding to the two different donors were clustered in the same branch, as it occurred with donors 1 and 4.

**FIGURE 2:**
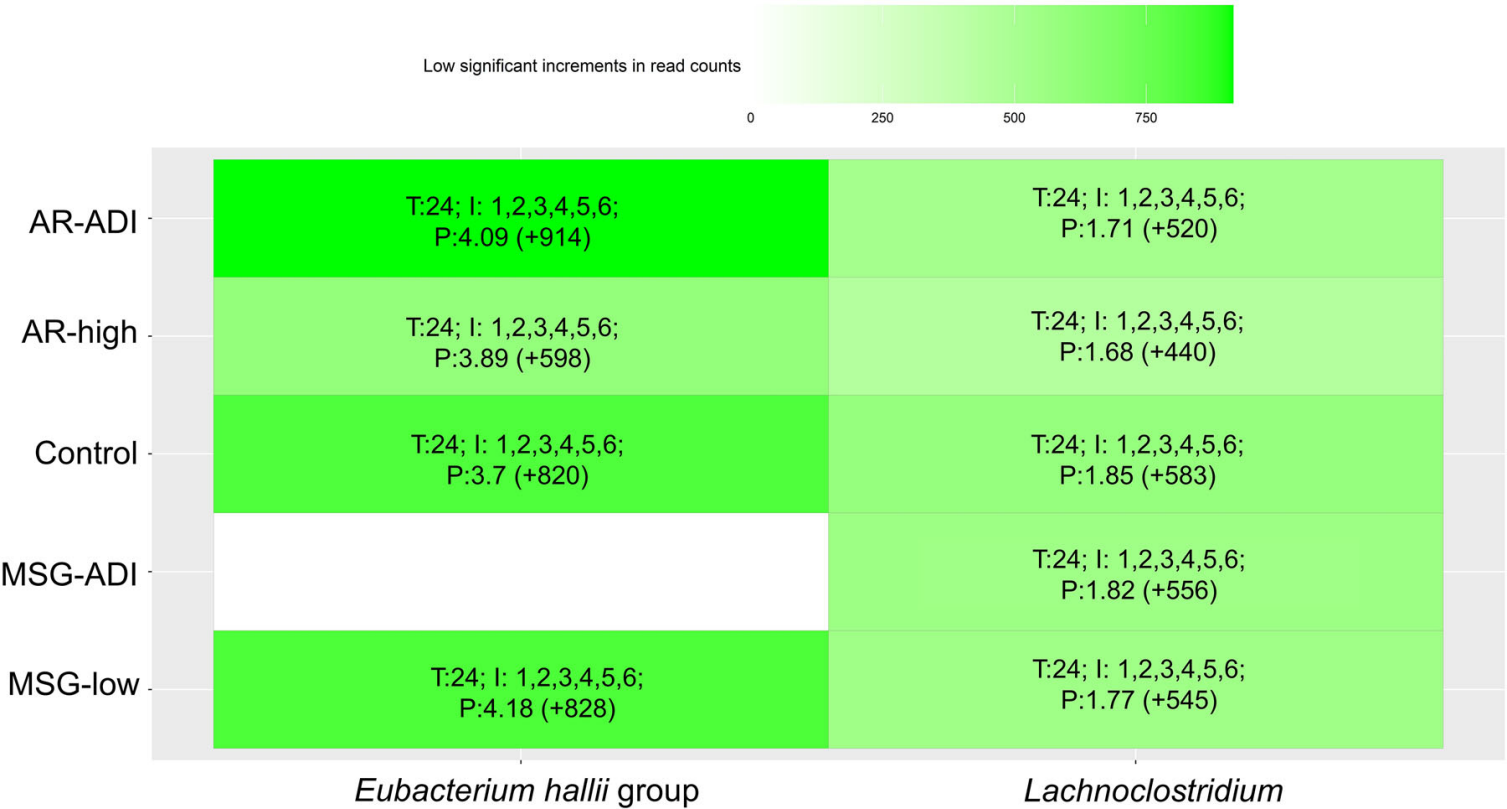
Statistically significant (*p_adj_* < 0.05) increments in bacterial genera after faecal fermentations of AR and MSG samples using faeces collected from healthy donors. AR and MSG concentrations were compared to controls (where no additive was added). T: fermentation time at which the maximum increment of a specific genus was observed. I: individuals (donors) showing the maximum increment of a genus in their microbiota. P: abundance percentage of a specific genus showing a maximum increment at a given time. Bacterial count increments are shown in parentheses.

A principal coordinate analysis (PCoA) of microbial taxa present in the microbiota of each donor and fermentation condition was computed to study further differences in microbiota profiles of samples (Supplementary Figure S4). Characteristic patterns could be found for each donor, yet some of them exhibited a higher degree of similarity between them, e.g. donors 1 and 4, and donors 3 and 6. Nevertheless, statistically significant differences (*p* < 0.05 and *padj* < 0.25) in individual taxonomic clades among donors and study conditions were observed using specific statistical methods tailored to analyze microbiota and microbiome data. In this sense, the greatest differences found in terms of bacterial taxa between the donors and between the fermentation and basal conditions are represented in Table 2, Supplementary and Supplementary Figure S5B. With regard to the comparison of the different donors, a high interindividual taxonomy variability was observed (Supplementary Figure S5A, Table 1), with no major effect of fermentation conditions on the beta diversity indices (Supplementary Figure S3). A characteristic microbiome pattern of all donors comprised three common majority genera (*Bacteroides, Bifidobacterium*, and *Blautia*). Then, a LMM was computed to further calculate microbial differences between groups of samples (Basal, Control, AR‐ADI, AR‐high, MSG‐ADI, and MSG‐low). Experimental groups and substrate doses were considered as fixed effects, while donor and sex were considered as random effects. Analysis of variance of random effects revealed a higher proportion of variance associated with donor (50–70%) than the sex (10–40%) random term. Regarding different fermentation conditions, it was remarkable the reduction in the relative abundance of *Prevotella* and *Faecalibacterium*, and the increase in the genus *Collinsella, Lachnoclostridium* and *Eubacterium hallii* group, in all fermented samples respect to the basal and irrespectively of the addition of the food additives (Supplementary Figure S5B; Table 2). It should be noted that *Eubacterium halii* and *Lachnoclostridium* showed statistically (*p* < 0.05 and padj < 0.25) significant increments during fermentation in all samples from all donors except MSG‐ADI, in which *Eu. halii* did not increase (Figure 2).

**TABLE 1:** Most abundant taxa that comprise the microbial profile of each donor at the basal level, expressed as abundance percentages (%).

Genus	Donor 1	Donor 2	Donor 3	Donor 4	Donor 5	Donor 6
*Dorea*	2.29	1.84	0.00	1.54	0.70	1.77
*Dialister*	5.59	0.01	1.98	2.80	0.00	1.97
*Coprococcus*	2.65	1.62	0.00	1.57	1.53	0.14
*Ruminococcus*	4.58	1.66	0.00	1.08	3.57	1.66
*Ruminococcus gauvreauii* group	0.93	0.17	0.00	0.19	0.05	0.02
*Lachnospiraceae* ND3007 group	1.04	0.57	0.00	0.53	0.31	0.35
*Collinsella*	3.73	6.05	0.00	4.93	4.42	0.00
*Subdoligranulum*	1.43	4.28	3.64	1.40	1.58	2.32
*Fusicatenibacter*	0.61	1.01	0.00	0.74	0.02	0.16
*Lachnospiraceae* FCS020 group	0.06	0.29	0.00	0.14	0.01	0.09
*Senegalimassilia*	0.27	0.66	0.00	0.42	0.00	0.00
*Blautia*	3.54	9.85	12.74	2.95	2.36	7.53
*Bacteroides*	0.74	14.83	36.07	2.01	25.71	25.65
*Faecalibacterium*	9.25	12.70	14.84	11.36	0.17	3.14
*Anaerostipes*	0.10	0.68	3.22	0.63	0.78	0.66
*Parabacteroides*	0.69	2.38	4.42	0.80	3.93	2.00
*Sutterella*	0.27	1.36	4.16	0.60	0.17	0.02
*Ruminococcus torques* group	1.01	1.34	2.91	0.31	2.20	1.20
*Butyricicoccus*	0.15	0.17	0.68	0.66	0.02	0.32
*Odoribacter*	0.05	0.47	0.66	0.08	0.57	0.29
*Bifidobacterium*	13.69	4.83	10.12	14.11	7.36	13.90
*Prevotella*	36.69	17.95	0.00	40.18	0.07	0.00
*Roseburia*	0.60	0.15	1.23	1.42	0.48	0.12
*Streptococcus*	0.17	1.61	0.16	1.68	0.05	0.74
*Escherichia‐Shigella*	0.03	0.53	0.00	0.00	5.99	0.90
*Intestinibacter*	0.31	0.36	0.29	0.23	3.42	0.46
*Romboutsia*	1.84	1.55	0.04	3.28	7.17	3.02
UCG‐002	0.79	1.99	0.04	0.41	2.77	0.61
UCG‐003	0.17	0.22	0.00	0.22	0.54	0.00
*Christensenellaceae* R‐7 group	0.31	0.34	0.00	0.12	6.17	0.35
UCG‐005	0.09	0.96	0.00	0.12	2.99	0.16
Family XIII AD3011 group	0.21	0.24	0.02	0.07	1.15	0.45
*Methanobrevibacter*	0.75	0.00	0.00	0.36	0.00	4.96
*Eubacterium hallii* group	0.77	1.52	0.26	1.79	2.48	2.96
*Erysipelatoclostridium*	0.00	0.18	0.21	0.00	0.05	2.12
*Alistipes*	0.33	3.95	0.21	0.00	4.95	5.59
CAG‐352	2.64	0.00	0.00	0.00	2.99	3.22
*Lachnoclostridium*	0.32	0.12	0.31	0.08	0.15	0.33
*Agathobacter*	0.64	0.22	0.00	0.89	0.45	3.85
*Oscillibacter*	0.00	0.31	0.04	0.00	0.31	0.95
*Barnesiella*	0.04	0.00	0.68	0.01	0.47	2.60
NK4A214 group	0.23	0.64	0.00	0.09	0.80	0.81
*Clostridium innocuum* group	0.00	0.03	0.55	0.00	0.23	1.57

**TABLE 2:** Most abundant taxa found in the microbiota in the presence and/or absence of MSG and AR, expressed as mean ± standard deviation (SD) of abundance percentages (%).^a,b^ Statistically significant (*p* < 0.05 adjusted by Holm method) differences between groups. To calculate these differences between groups of samples (Basal, Control, AR‐ADI, AR‐high, MSG‐ADI, and MSG‐low), a LMM was computed. Experimental groups and substrate doses were considered as fixed effects while donor and sex were considered as random effects.

Genus	Basal Mean ± SD	Control Mean ± SD	AR‐ADI Mean ± SD	AR‐high Mean ± SD	MSG‐ADI Mean ± SD	MSG‐low Mean ± SD
*Prevotella*	15.82 ± 18.88^a^	9.76 ± 12.29^b^	8.91 ± 11.31 ^b^	9.55 ± 11.89 ^b^	8.80 ± 11.82 ^b^	9.45 ± 11.85 ^b^
Other	9.31 ± 5.03 ^a^	8.11 ± 2.96 ^a^	9.21 ± 4.37 ^a^	9.13 ± 5.25 ^a^	8.76 ± 4.51 ^a^	8.76 ± 4.33 ^a^
*Faecalibacterium*	8.58 ± 5.74 ^a^	3.24 ± 2.44 ^b^	2.96 ± 2.17 ^b^	3.32 ± 2.68 ^b^	2.76 ± 2.04 ^b^	2.96 ± 2.14 ^b^
*Alistipes*	2.51 ± 2.60 ^a^	2.29 ± 1.76 ^a^	2.15 ± 1.50 ^a^	1.98 ± 1.48 ^a^	2.34 ± 1.65 ^a^	2.22 ± 1.96 ^a^
*Dialister*	2.06 ± 2.07 ^a^	1.81 ± 1.46 ^a^	1.73 ± 1.51 ^a^	1.73 ± 1.56 ^a^	1.70 ± 1.46 ^a^	1.72 ± 1.45 ^a^
*Ruminococcus*	2.09 ± 1.68 ^a^	1.44 ± 1.55 ^a^	1.26 ± 1.31 ^a^	1.24 ± 1.26 ^a^	1.26 ± 1.18 ^a^	1.31 ± 1.45 ^a^
*Subdoligranulum*	2.44 ± 1.24 ^a^	1.28 ± 1.00 ^b^	1.19 ± 0.91 ^b^	1.14 ± 1.01 ^b^	1.18 ± 0.93 ^b^	1.27 ± 1.05 ^b^
*Romboutsia*	2.82 ± 2.43 ^a^	1.02 ± 0.70 ^b^	0.94 ± 0.60 ^b^	1.00 ± 0.61 ^b^	0.99 ± 0.63 ^b^	0.99 ± 0.68 ^b^
[*Ruminococcus*] *torques* group	1.50 ± 0.92 ^a^	0.98 ± 0.51 ^a^	1.15 ± 0.97 ^a^	1.18 ± 1.01 ^a^	1.02 ± 0.51 ^a^	1.03 ± 0.74 ^a^
CAG‐352	1.48 ± 1.63 ^a^	0.77 ± 0.85 ^b^	0.57 ± 0.65 ^b^	0.71 ± 0.78 ^b^	0.67 ± 0.74 ^b^	0.77 ± 0.85 ^b^
*Bacteroides*	17.50 ± 14.19 ^a^	17.51 ± 11.70 ^a^	16.70 ± 11.98 ^a^	16.46 ± 11.64 ^a^	17.17 ± 11.73 ^a^	17.14 ± 12.05 ^a^
*Methanobrevibacter*	1.01 ± 1.96^b^	3.35 ± 3.99^a^	2.74 ± 3.19ª^,b^	2.55 ± 2.85ª^,b^	2.57 ± 3.18ª^,b^	3.25 ± 3.79^a^
*Lachnoclostridium*	0.22 ± 0.011 ^b^	1.85 ± 1.22^a^	1.71 ± 1.16 ^a^	1.68 ± 0.83 ^a^	1.82 ± 1.12 ^a^	1.77 ± 0.97 ^a^
*Fusicatenibacter*	0.42±0.042 ^a^	0.72 ± 0.91 ^a^	0.63 ± 0.69 ^a^	0.54 ± 0.59 ^a^	0.59 ± 0.63 ^a^	0.59 ± 0.69 ^a^
*Bifidobacterium*	10.67 ± 3.92 ^a^	11.00 ± 5.18 ^a^	11.90 ± 5.18 ^a^	11.20 ± 4.42 ^a^	11.58 ± 5.39 ^a^	11.03 ± 4.57 ^a^
*Intestinibacter*	0.85 ± 1.26 ^a^	1.42 ± 1.12 ^a^	2.17 ± 3.13 ^a^	1.52 ± 1.46 ^a^	1.40 ± 1.49 ^a^	1.69 ± 2.31 ^a^
*Coprococcus*	1.25 ± 1.01 ^a^	1.19 ± 0.94 ^a^	1.40 ± 1.09 ^a^	1.24 ± 0.99 ^a^	1.34 ± 1.11 ^a^	1.24 ± 0.99 ^a^
*Blautia*	6.49 ± 4.24 ^a^	6.49 ± 3.29 ^a^	6.82 ± 3.30 ^a^	7.27 ± 3.42 ^a^	6.75 ± 4.24 ^a^	6.56 ± 3.35 ^a^
*Collinsella*	3.19 ± 2.58 ^b^	6.18 ± 5.56 ^a^	6.51 ± 5.46 ^a^	7.10 ± 6.28 ^a^	6.20 ± 5.36 ^a^	6.29 ± 5.34 ^a^
UCG‐002	1.10 ± 1.05 ^b^	1.68 ± 0.95 ^a,b^	1.87 ± 1.10 ^a^	1.94 ± 1.11 ^a^	1.81 ± 1.08 ^a^	1.82 ± 1.03 ^a^
*Escherichia‐Shigella*	1.24 ± 2.36 ^b^	5.39 ± 8.07 ^a^	4.61 ± 7.01 ^a,b^	4.96 ± 7.80 ^a^	5.40 ± 7.85 ^a^	4.81 ± 7.39 ^a,b^
*Parabacteroides*	2.37 ± 1.55 ^b^	3.42 ± 1.81 ^a^	3.14 ± 1.45 ^a,b^	3.31 ± 1.28 ^a^	3.53 ± 1.46 ^a^	3.50 ± 1.60 ^a^
*Dorea*	1.36 ± 0.85 ^b^	2.19 ± 1.87 ^a,b^	2.38 ± 2.11 ^a,b^	2.19 ± 1.68 ^a,b^	2.51 ± 1.96 ^a^	2.30 ± 1.86 ^a,b^
*Anaerostipes*	1.01 ± 1.11 ^b^	1.65 ± 1.93 ^a,b^	1.80 ± 1.78 ^a,b^	1.79 ± 1.01 ^a,b^	1.90 ± 1.76 ^a^	1.50 ± 1.40 ^a,b^
[*Eubacterium*] *hallii* group	1.63 ± 1.01 ^b^	3.70 ± 1.51 ^a^	4.09 ± 2.17 ^a^	3.89 ± 1.96 ^a^	4.11 ± 1.97 ^a^	4.18 ± 1.90 ^a^
*Sutterella*	1.10 ± 1.57 ^b^	1.56 ± 2.09 ^a,b^	1.49 ± 1.79 ^a,b^	1.40 ± 1.62 ^a,b^	1.83 ± 2.31 ^a^	1.85 ± 2.39 ^a^

### 3.2 Metabolites Determination

**FIGURE 3:**
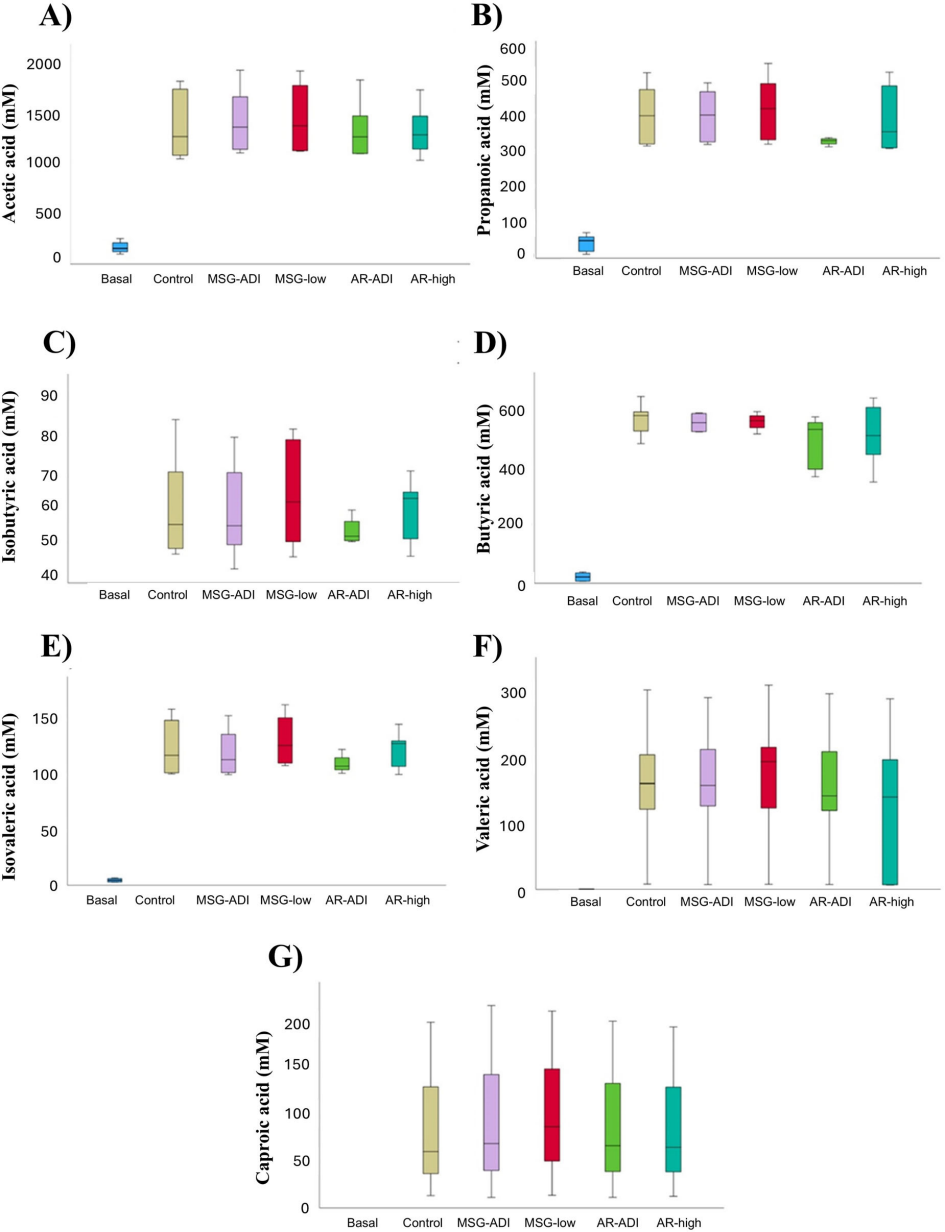
Levels (mM) of SCFAs determined by GC in the cell‐free supernatants of the six faecal donors used in this study. The grey bar represents the mean (and standard deviation) of the six donors. The basal (0 h) and control (24 h) samples correspond to the concentration of SCFAs present in the faeces of the donors, i.e. without the addition of an additive. The panels show the different values obtained for SCFAs: acetic acid (A), propanoic acid (B), isobutyric acid (C), butyric acid (D), isovaleric acid (E), valeric acid (F), and caproic acid (G).

In relation to SCFA production, the levels of acetic, butyric, isobutyric, propanoic, valeric, and isovaleric acids were significantly higher (*p <* 0.05) in faecal fermentations as compared to basal donor samples. Interestingly, the values obtained for the control fermentations were in the same range as those obtained in the samples fermented in the presence of the different additives/doses and much higher than those detected in basal samples (Figure 3, Supplementary Table S2). This demonstrates the metabolic activity of the microbiota in all the tested conditions and could indicate that short‐term exposure to the different concentrations of the food additives analyzed have no major influence on the human gut microbial metabolic activity in terms of the production of saccharolytic end products (SCFAs).

**FIGURE 4:**
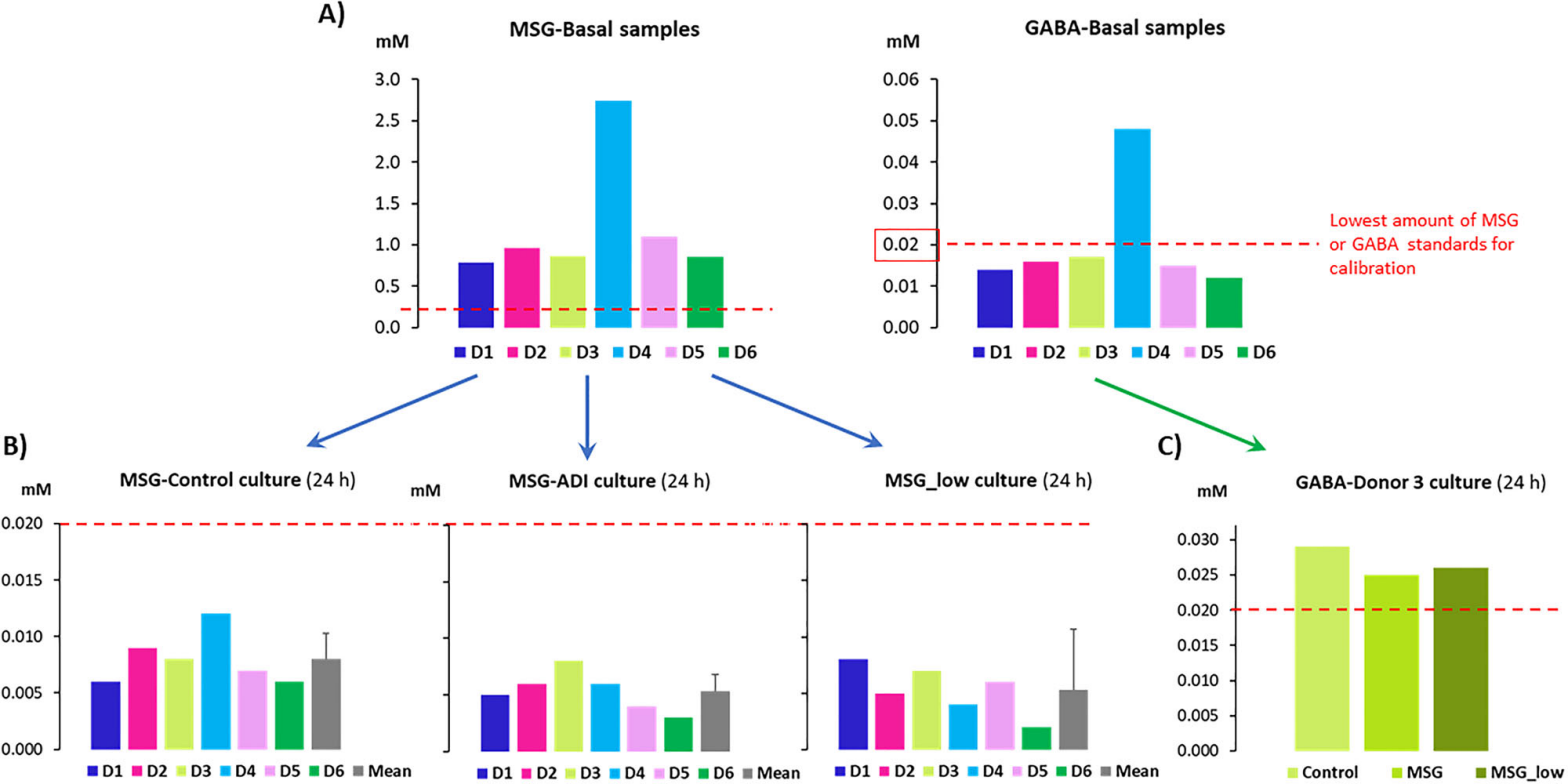
Levels (mM) of MSG and its microbial‐derivate metabolite GABA determined by LC/PDA in the cell‐free supernatants of the six faecal donors used in this study. The grey bar represents the mean (and standard deviation) of the six donors. The basal (0 h) and control (24 h) samples correspond to the concentration of MSG and GABA present in the faeces of the donors, i.e., without the addition of MSG (A). The values in the cultured (24 h) MSG and MSG‐low correspond to the samples initially supplemented with 0.8 and 0.08 mM of MSG, respectively (B). GABA in the samples cultured for 24 h was only detected in donor 3 (C).

In addition, levels (mM) Level of relevant metabolites derived from the microbial transformation of either MSG or AR was performed. The MSG concentration in the basal faecal samples showed similar values for all donors, except donor 4 that presented about three‐times higher concentrations than the others. However, levels of faecal GABA were below the lowest concentration of the standard used for calibration, which is denoted with a red line in Figure 4A (Supplementary Table S2). Concentrations of both MSG and GABA decreased after 24 h of fermentation in all the conditions tested (control, MSG‐low and MSG‐ADI), being both below the lowest concentration of both standards (Figure 4B). The only exception was donor 3, in which GABA production was observed in comparison to its basal sample (Figure 4C).

Determination of standards of AR and derived metabolites (i.e., 4‐amino‐5‐methoxy‐2‐methylbenzenesulfonic acid (4A5M2M) and 1‐amino‐2‐naphthol‐6‐sulfonic acid (1A2N6S)) in acetonitrile solutions was successfully accomplished by LC‐MS (Supplementary Figure S6A). However, only the 4A5M2M metabolite could be detected and quantified in the tested samples, unlike the 1A2N6S metabolite. Further experiments demonstrated the instability of the latter metabolite in the BFM since the 1A2N6S standard was readily unstable in water (Supplementary Figure S6B) and, therefore, it would be also plausible its fast degradation in an *in* *vitro* faecal environment immediately after its potential formation.

Strikingly, a partial degradation of AR to the 4A5M2M metabolite was observed when AR was added to the BFM and autoclaved before starting faecal fermentation (Supplementary Figure S7). Moreover, AR was completely metabolized following faecal fermentation since no traces of the intact food additive remaining after the autoclaving could be detected in all fermented samples (high and ADI doses) (Supplementary Figure S8), suggesting the microbial transformation of AR by gut bacteria. Finally, levels of faecal 4A5M2M were remarkably lower, regardless of the donor, in the AR‐ADI dose samples (ranging from 0.015 to 0.025 mM, mean value of 0.021 mM) than in the AR‐high dose samples (ranging from 0.27 to 0.39 mM, mean value of 0.31 mM). Moreover, the difference in 4A5M2M concentration determined in the AR‐ADI and AR‐high dose fermented samples was fairly proportional to the different levels of starting AR concentrations used in both doses (i.e., a 10‐fold difference) (Table S2).

### 3.3 Correlation Between Microbiota Profiling and Metabolites

**FIGURE 5:**
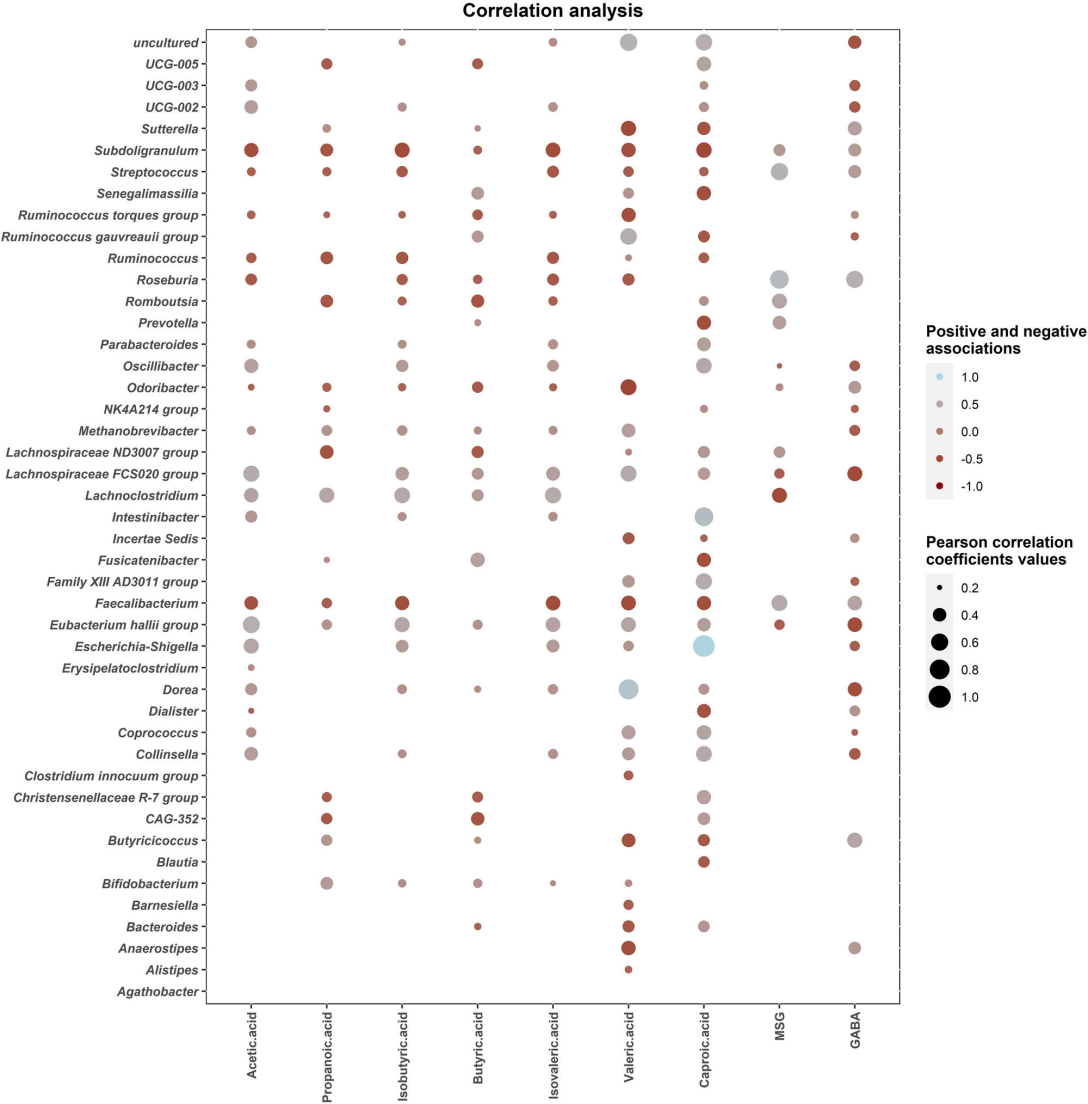
Heat map showing Pearson's correlation between significant taxa and SCFAs, GABA, and MSG values, and microbial counts in different culture media. Red and blue points indicate positive and negative correlations, respectively, whereas point sizes indicate the Pearson correlation coefficient values. Color intensity and point sizes are in proportion to the magnitude.

To gain a better understanding of the dynamics of the faecal cultures, statistical correlations between SCFA levels and microbial clades modulated by MSG and AR addition were determined (Figure 5). As shown, *Escherichia*‐*Shigella*, *Eubacterium halii*, *Lachnospiraceae* FCS020 group, and *Lachnoclostridium* showed positive correlations with the major SCFAs acetic, propanoic, isobutyric, butyric isovaleric, valeric, and caproic acids. The strongest positive correlations were found between *Dorea* and valeric acid, *Escherichia*‐*Shigella* and *Intestinibacter* with caproic acid, and *Roseburia* and *Streptococcus* with GABA. On the contrary, many negative correlations were found, such as *Roseburia* and butyric acid. Correlations between microbial profiles and AR and its derived metabolites, 4A5M2M and 1A2N6S, were not conducted as most of these metabolites appeared to be generated during the heat treatment of the media rather than by microbial transformation as previously described.

### 3.4 Cytotoxicity of Supernatants From Faecal Cultures

The potential cytotoxic effect of the two food additives, as well as the derived metabolites obtained by microbiota transformation, was *in* *vitro* analyzed in the HT29 cell line. The continuous monitoring of the cell index after addition of MSG and AR at the highest concentration tested (0.8 and 0.6 mM, respectively) showed no drastic drops. This suggests that both additives directly added to the cell line did not have a cytotoxic effect (Supplementary Figure S9A, B). On the other hand, when HT29 monolayers were in contact with cell‐free supernatants obtained from faeces of the six donors, either the basal samples (0 h) or control samples cultivated for 24 h, and also in all concentrations tested for both additives, no variations in the monitored cell index were observed (Supplementary Figure S9C). Graph representation of cell index normalized by the basal line (at time 0), which allows a better comparison of samples, showed similar normalized‐CI, with a tendency to increase with time, than those obtained with the reference base‐line calculated in wells with HT29 cells not exposed to any challenge (Supplementary Figure S9D). These results show that the HT29 monolayers remained intact in all conditions tested and, therefore, the food additives MSG and AR added had no cytotoxic effect under our experimental conditions.

## 4 Discussion

Several studies have suggested a certain degree of interaction between food additives and gut microbiota, although substantial efforts still need to be taken to comprehensively assess whether food additives such as MSG and AR may affect gut microbiota and human health (Chassaing et al. 2022; He et al. 2021; FAO/WHO 2016; Mercier‐Bonin et al. 2018; Cao et al. 2020; Zhou et al. 2023). Results from our approach in vitro with human faecal microbiotas were consistent with those previously described in the literature and demonstrate that the individual food additives at the tested doses caused no remarkable effects on the gut microbiota composition and activity in *in* *vitro* batch faecal culture models, as compared to control fermentations conducted in the absence of any food additive. Remarkably, although alpha and beta‐diversity analysis demonstrated certain interindividual variability in microbiota profiles in agreement with prior results (Kruger et al. 2024), the response to the different additives and doses followed similar patterns in all individuals, with limited effects on the microbiota.

MSG, used as flavour enhancer in some food products, did not cause any significant variations in the community composition, as compared to samples fermented without food additives, although available literature reported that some taxa tended to change in the presence of certain food additives. A previous study in pigs reported that MSG promoted the colonization of microbes related to energy extraction in the gut, such as *Faecalibacterium prausnitzii* and *Roseburia* (Feng et al. 2015). With regard to human gut microbiota, it has been described a slight decrease in the content of the phylum *Bacillota* as the intake of MSG increased gradually, while the content of *Actinomycetota* (former *Actinobacteria)* increased slightly during the MSG consumption. Moreover, a slight decrease of *Faecalibacterium*, *Megamonas*, and *Blautia* with the consumption of MSG was observed, whereas the content of *Collinsella* increased (Peng et al. 2018). However, in our study *in* *vitro*, no changes were found for these species in response to the different additives.

On the other hand, no significant changes were detected in the microbial composition for fermentations in the presence of AR, although a slight increase in the abundance of *Intestinibacter* was detected in the AR‐ADI dose samples, compared to the basal, control and MSG samples. However, the effects of AR on the human gut microbiota have not yet been studied, although it is known that the effect of other food colorants has little impact on gut microbiota (Pinget et al. 2019).

Differences in the results from our investigation as compared to those previously reported in the literature may be related to differences in the investigation models and/or the basal microbiota configuration of the faecal donors. In relation to the models for investigation, herein we have employed an *in* *vitro* faecal fermentation model that allows monitoring the response of one individual's microbiota to various doses/ingredients, facilitating the separation of the effect that the ingredient under investigation may have from interferences with other dietary ingredients that may occur in human intervention trials. Regardless of these advantages, this model has limited performance to conduct long‐term investigations, or to mimic complex dietary patterns. Our results could only detect minor gut microbiota modulatory effects of these additives, yet they were consistent across the different faecal donors. In relation to interindividual variability in the basal microbiota configuration of the faecal donors, which could be related to different dietary patterns, it has been described as an important source of variability differentiating responders and non‐responders to a given ingredient. Herein we have performed the investigation with a limited number of donors without collecting their dietary habits, as an exploratory preliminary investigation. However, ruling out potential negative impacts of the tested additives on the microbiota may need further investigation with more complex models mimicking interaction of the additives with other dietary ingredients, modeling the impact of long‐term chronic consumption and a larger number of faecal donors, accounting for a potential individual response to dietary ingredients.

Additionally, SCFAs, which are produced by gut bacteria through saccharolytic fermentation of complex resistant carbohydrates, are important metabolites that influence host health (Xie et al. 2017; Richards et al. 2016). Our results show that the administration of MSG and AR does not seem to affect SCFA production, as would be expected since the microbial populations are not drastically altered. Specifically, major SCFA producers such as *Prevotella*, *Ruminococcus*, *Bifidobacterium*, *Firmicutes*, *Bacteroides*, *Faecalibacterium prausnitzii*, and members of the family *Lachnospiraceae*, among others, did not show alterations in the presence of either MSG or AR.

Finally, we have observed a tendency towards MSG and GABA decrease after faecal cultures with respect to the basal samples in most donors, although a small amount of GABA was detected in cultures from one particular donor after fermentation (donor 3, basal amount 0.017 mM; fermented cultures: 0.029 mM, 0.025 mM and 0.026 mM, for control, MSG‐ADI and MSG‐low samples, respectively). MSG and GABA decrease along the fermentations could be due to either consumption or transformation into alternative metabolites. However, it is worth highlighting that the detected concentrations of both MSG and GABA in most samples are very low and fall under the detection limit of the technique used, hence the interpretation of the tendencies needs to be taken with caution. Interestingly, some species of the gut microbiome can convert dietary glutamate to GABA including, *Bacteroides*, *Parabacteroides*, *Bifidobacterium*, and *Escherichia* species as previously reported (Duranti et al. 2020; Strandwitz et al. 2019). The presence of *Bacteroides* could be the reason why GABA production is observed only in the cultures from donor 3, which also exhibits the highest relative abundance of this taxa. The no detection of GABA in cultures from the other donors could be attributed to the possible presence of GABA‐consuming microorganisms as previously reported (Strandwitz et al. 2019), or to the lowest representation and metabolic activity of GABA producing taxa.

Regarding AR‐derived metabolites, 4A5M2M was found in all fermented AR‐ADI and AR‐high dose samples at different levels as a function of the starting AR doses. This evidence could point towards bacterial‐mediated transformations of AR by the microbial communities present in the human gut microbiome in agreement with previous *in* *vitro* and *in* *vivo* findings that described a biotransformation pathway, involving azo‐reductases expressed by gut bacteria, into sulfonated aromatic amines, as the main mechanism of metabolization of AR (He et al. 2021; Zou et al. 2020). However, according to our observations, we cannot uniquely ascribe the conversion of AR to 4A5M2M to gut microbial biotransformation, as our results also revealed the partial instability of AR to a severe heat treatment like autoclaving (121°C for 15 min) and the contribution of the processing to the conversion of AR to sulfonated aromatic amines such as 4A5M2M. This unexpected observation may have relevant implications from a food safety perspective and deserves further attention, as it indicates that thermal processing that may occur during food processing, manufacturing, and consumption can affect the chemical structure of food additives. The presence of AR‐derivatives generated during food processing in AR‐containing foods and the possible risks these transformations may pose on the consumer should be further evaluated. Besides, the observed instability in water solutions of the other main sulfonated aromatic amine (i.e., 1A2N6S) (Supplementary Figure S6B) impaired its detection in the faecal samples. Zou et al. (2020) investigated the potential impact of some azo‐dye colorants used as excipients on intestinal drug absorption. This study showed that human gut bacteria belonging to the *Bacteroidota* (e.g., *Bacteroides fragilis, B. vulgatus, Odoribacter splanchnicus*) and *Bacillota* (e.g., *Clostridium ramosum, C. hylemonae, Faecalicoccus pleomorphus*) phyla are the most active metabolisers of AR. He et al. (2021) reported that AR and Yellow 6 sunset yellow (E110) were metabolized by azo‐reductases expressed by commensal bacteria, identified as *Bacteroides ovatus* and *Enterococcus faecalis*, of the mice gastrointestinal tract into these two sulfonated aromatic amines. In addition, the intake of these azo‐dye colorants promoted colitis in these animals with dysregulated expression of the pro‐inflammatory interleukin‐23 cytokine. Interestingly, AR‐induced colitis was partly dependent on the microbiota, although it did not depend on marked shifts in its composition because no significant changes were observed in the faecal bacterial composition, which is in line with the findings described in our work. These authors also indicated that it is unclear if sulfonated aromatic amines are further processed and bind bacterial or host proteins to induce an immune response.

On the other hand, food additives are widely used in food manufacturing to improve properties such as colour, taste, smell, nutritional value, and shelf life of food products (EFSA ANS Panel 2015; EFSA ANS Panel 2017; FAO/WHO 2016). Within the formulation process, these additives may undergo thermal processing, such as sterilization or pasteurization, to eliminate heat‐resistant pathogens and spoilage organisms (Tadini and Gut 2022); thermal preparation unit conditions such as texturization, shearing, gelatinization, extrusion, blanching, dehydration, etc., (Shelar and Gaikwad 2019; Sule et al. 2024), or thermal cooking in household settings, where temperatures above 100°C (121–163°C) must be reached for a defined period depending on the product and treatment (Preethi et al. 2012; Nwosu et al. 2014; Chiozzi et al. 2022). In order to replicate either of the processes mentioned above, which might include potential additives like MSG for flavoring or AR for coloring, these additives were autoclaved along with the BFM prior to the *in* *vitro* fermentation. As shown above, in the case of AR, a partial degradation to the 4A5M2M metabolite was observed due to the autoclaving, that warrants further investigation into the effect of processing on the integrity of certain food additives, as well as the potential consequences of their safety profile.

As far as we could find, no literature about the effect of MSG or AR on intestinal cellular lines was reported. Under our experimental conditions, using an *in* *vitro* model of HT29, no cytotoxic effect of both additives at the doses tested was observed. This could be an expected result given that the doses tested were close, or below, to the ADI recommended by EFSA in both cases.

In conclusion, our study suggests that individual MSG and AR, at the tested doses and under our experimental conditions, do not appear to have significant effects directly on the taxonomical composition of the microbial communities present in the human gut, nor on the host intestinal cells. However, we acknowledge that the investigation has certain limitations such as the low number of donors, lack of chronic exposure, lack of *in* *vivo* experiments, and lack of interaction with different food additives and/or dietary patterns. Investigation with a larger number of faecal donors accounting for different dietary patterns may help elucidate whether potential effects of food additives on the microbiome may be dependent on specific gut microbiome configurations. The possible microbial‐mediated transformation of MSG and AR under faecal fermentation conditions needs to be further confirmed. The possible effects produced by the metabolites generated from their degradation, either exerted by the gut microbiota or heat‐based processing should be further investigated.

## Author Contributions

**Adela Granja‐iglesias**: investigation, writing – original draft, writing – review and editing. **Xenia Vázquez**: investigation, writing – original draft, writing – review and editing, software, formal analysis. **Carlos Sabater**: investigation, writing – original draft, software, formal analysis, writing – review and editing. **Arancha Hevia**: investigation, writing – review and editing. **Manuel Garrido‐romero**: investigation, methodology, writing – review and editing. **Ana Muñoz‐labrador**: investigation, methodology, writing – review and editing. **Plácido Galindo‐iranzo**: investigation, methodology, writing – review and editing. **Rosa Lebrón‐aguilar**: investigation, methodology, writing – review and editing. **Jesús E. Quintanilla‐lópez**: investigation, methodology, writing – review and editing. **F. Javier Moreno**: conceptualization, investigation, funding acquisition, writing – original draft, project administration, resources, supervision. **Lorena Ruiz**: conceptualization, investigation, funding acquisition, writing – original draft, project administration, supervision, resources. **Patricia Ruas‐madiedo**: conceptualization, investigation, funding acquisition, writing – original draft, project administration, supervision, resources.

## Conflicts of Interest

The authors declare no conflicts of interest.

## Supporting information




**Supplementary Figures** reporting the evolution of PH and viable bacteria during the fermentation experiments (Supplementary Figure S1).
Alpha‐diversity and beta‐diversity indicators, and principal coordinate analyses in the different fecal fermentations (Supplementary Figures S2, S3,S4 and S5).
SCFA concentrations determined in all the fermentation conditions (Supplementary Table S1).
Representative chromatograms showing the stability of a mixture of AR and its derived metabolite (Supplementary Figure S6), the impact of autoclaving on AR (Supplementary Figure S7) and the representation of AR and its derived metabolite, 4A5M5M, in the fecal fermentation media.
