# Supplementary material for: In Vitro Investigation of the Effects of the Food Additives Monosodium Glutamate and Allura Red AC on the human Gut Microbiota and Intestinal Cell Lines
Source: J Food Sci. 2025 Aug 24;90(8):e70504. doi: 10.1111/1750-3841.70504 (PMC12375840; doi:10.1111/1750-3841.70504)
Supplement: Supplementary file 1 — Supplementary Figures reporting the evolution of PH and viable bacteria during the fermentation experiments (Supplementary Figure S1). Alpha‐diversity and beta‐diversity indicators, and principal coordinate analyses in the different fecal fermentations (Supplementary Figures S2, S3,S4 and S5). SCFA concentrations determined in all the fermentation conditions (Supplementary Table S1). Representative chromatograms showing the stability of a mixture of AR and its derived metabolite (Supplementary Figure S6), the impact of autoclaving on AR (Supplementary Figure S7) and the representation of AR and its derived metabolite, 4A5M5M, in the fecal fermentation media. [file JFDS-90-0-s001.docx]

**Food additives monosodium glutamate and Allura red AC show limited effect on the human gut microbiota and intestinal cell lines using *in vitro* models**

Adela Granja-Iglesias^1,2&^, Xenia Vázquez^1,2&^, Carlos Sabater^1,2^, Arancha Hevia^1,2^, Manuel Garrido-Romero^3^, Ana Muñoz-Labrador^3^, Plácido Galindo-Iranzo^4^, Rosa Lebrón-Aguilar^4^, Jesús E. Quintanilla-López^4^, F. Javier Moreno^3^*, Lorena Ruiz^1,2^*, Patricia Ruas-Madiedo^1,2^

**^1^** Group of Functionality and Ecology of Beneficial Microorganisms (MicroHealth), Dairy Research Institute of Asturias (IPLA-CSIC), Paseo Río Linares s/n, 3300, Villaviciosa, Asturias, Spain.

**^2^** Health Research Institute of Asturias (ISPA), Avenida Hospital Universitario s/n, 33011, Oviedo, Asturias, Spain.

**^3^** Group of Chemistry and Functionality of Carbohydrates and Derivatives, Institute of Food Science Research, CIAL (CSIC-UAM), Nicolás Cabrera, 9, Campus de Cantoblanco, Universidad Autónoma de Madrid, 28049, Madrid, Spain.

^4^ Group of Photolysis and Chromatography, Institute of Physical Chemistry ‘Blas Cabrera’ (IQF-CSIC), Serrano, 119, 28006, Madrid, Spain.

^&^These authors have equally contributed to this work

***Keywords***: monosodium glutamate, Allura red AC, microbiota, food additives

***Running title***: Effect of food additives on microbiota and host

***Corresponding authors***: Javier Moreno ([javier.moreno@csic.es](mailto:javier.moreno@csic.es)) and Lorena Ruiz ([lorena.ruiz@ipla.csic.es](mailto:lorena.ruiz@ipla.csic.es))

**Supplementary Figure S1.** Values of pH (**A**) and viable cultivable bacteria (**B**) obtained in the basal sample (0 h) and in the five cultivation conditions, following 24 h incubation, for each of the 6 faecal donors. The average values (mean ± standard deviation) of both parameters is also presented (**C**). The doses of food additive used were: 0.8 mM for MSG-ADI, 0.08 mM for MSG-low, 0.06 mM for AR-ADI and 0.6 mM for AR-high.

**Supplementary Figure** **S2**. Comparison of different alpha-diversity indicators (Chao1, Shannon, Simpson and Inverse Simpson, based on taxonomic profiles). These indicators measure the variability of microbial taxa within a sample.

**Supplementary Figure S3.** Beta-diversity analysis of taxonomic profiles found in the microbiota of basal, control, AR-high, AR-ADI, MSG-low and MSG-ADI samples. Bray-Curtis method was selected for the calculation. ^a^ No statistically significant (p > 0.05 adjusted by Holm method) differences between groups were found. To perform the statistical analysis of different groups of samples (Basal, Control, AR-ADI, AR-high, MSG-ADI and MSG-low), a linear mixed-effects model (LMM) was computed. Experimental groups and substrate doses were considered as fixed effects while donor and sex were considered as random effects.


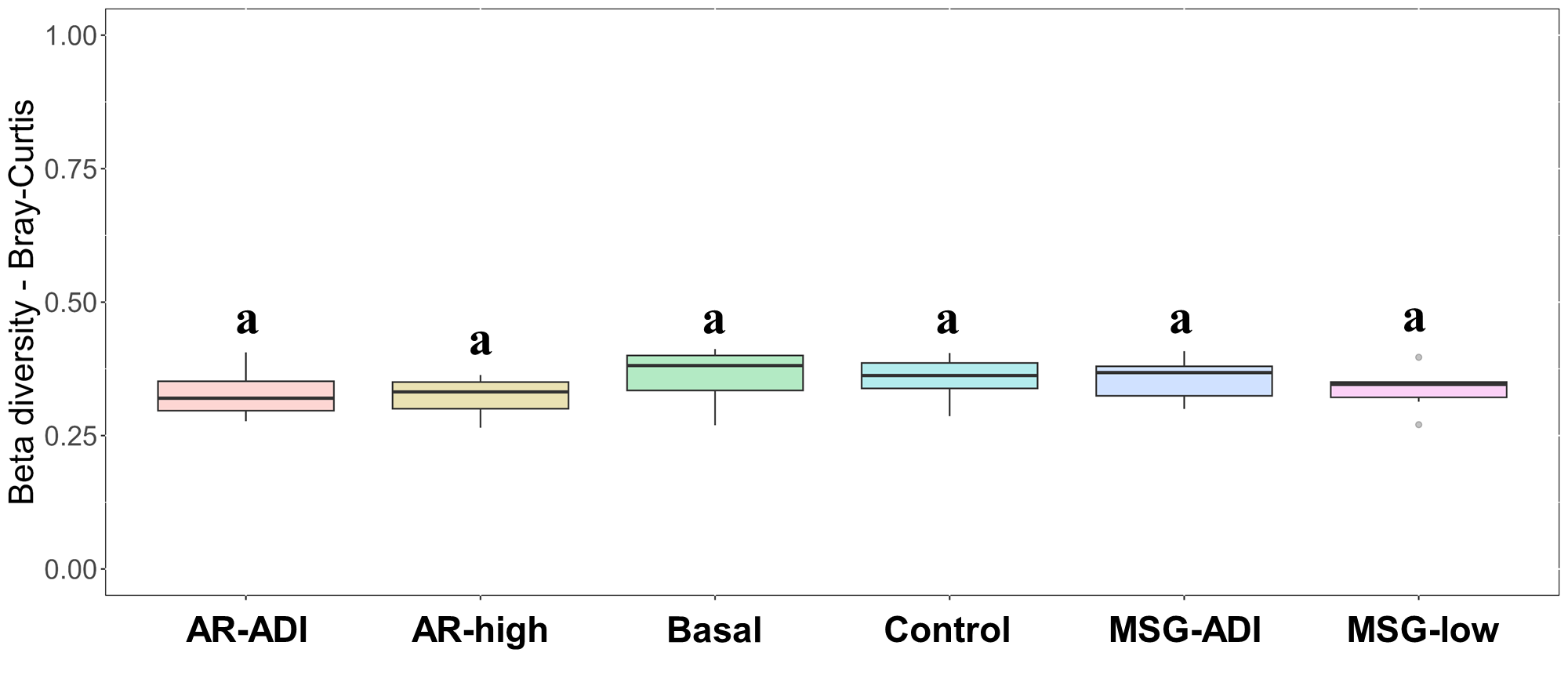


**Supplementary Figure S4**. Principal coordinates analysis (PCoA) of taxonomic profiles found in the microbiota of basal, control, AR-high, AR-ADI, MSG-low and MSG_ADI samples. PC: principal coordinate. The percentage of variance explained by each PC is indicated in the axis.


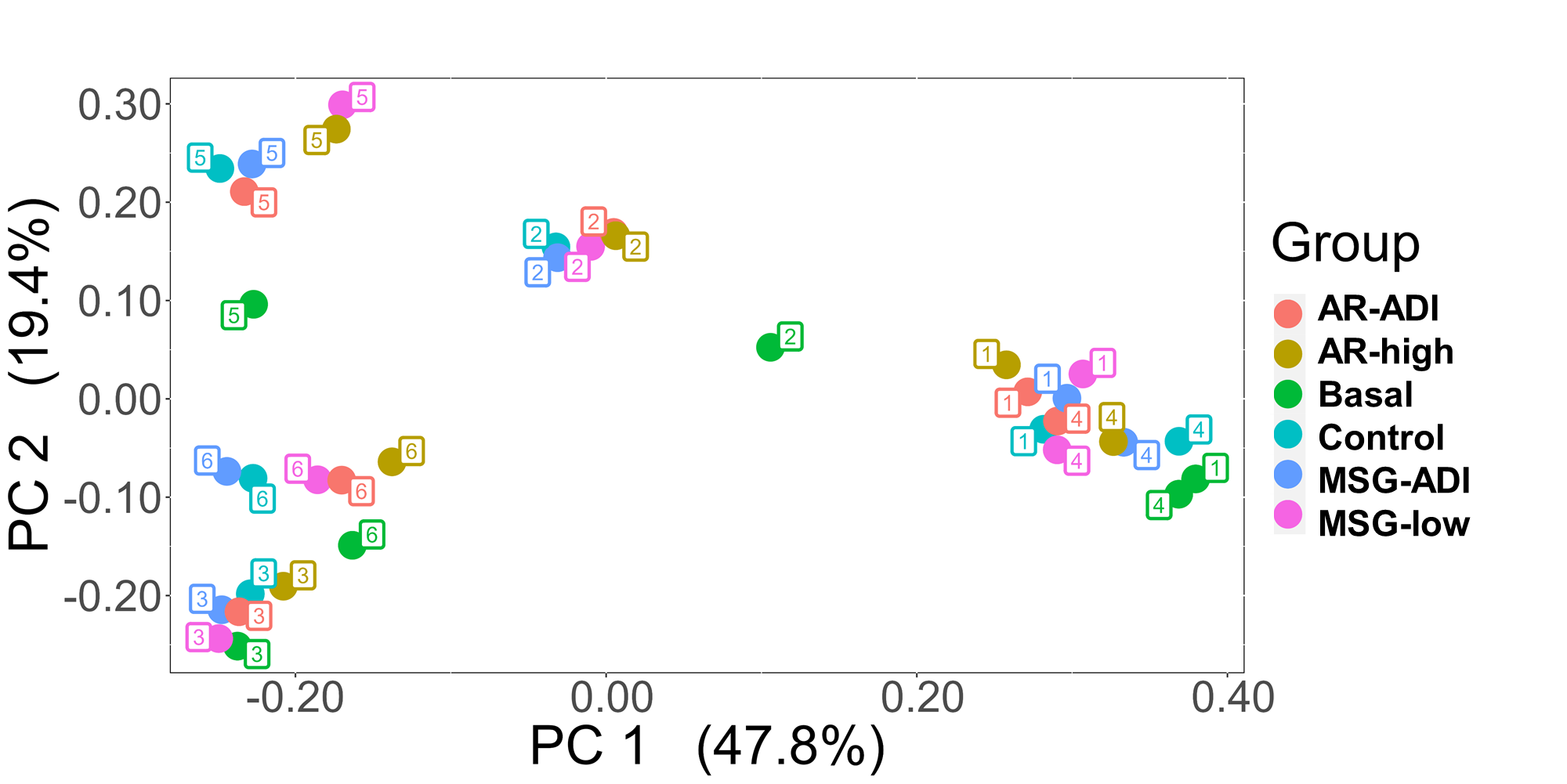


**Supplementary S5**. Representation of the relative abundance of most abundant taxa found in the microbiota composition: **A**) of basal samples of donors and **B**) in presence and/or absence of MSG and AR. These taxa constitute the core microbiota of individuals. Data are expressed as abundance percentages (%).


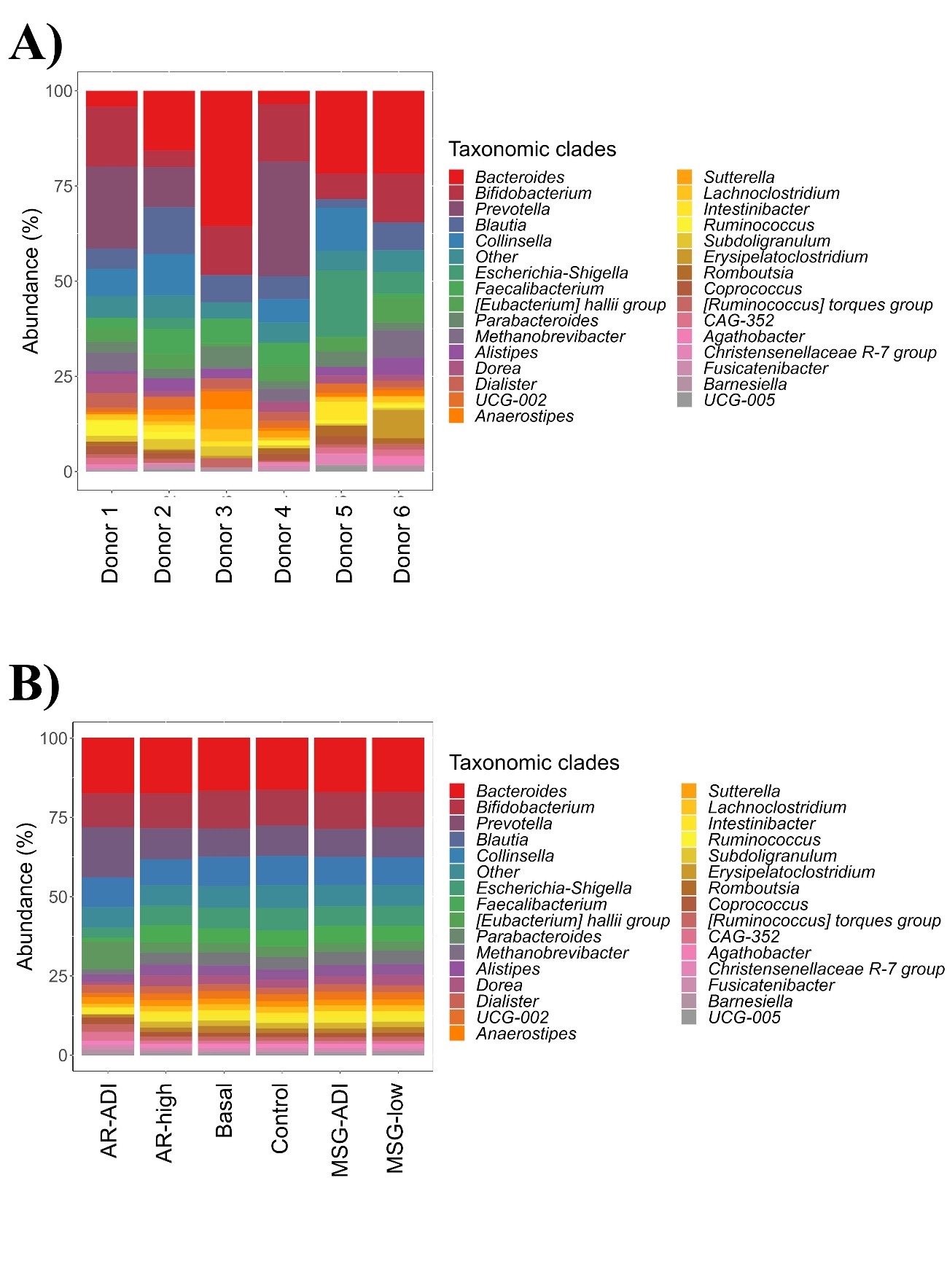


**Supplementary Figure S6.** LC-MS chromatograms AR-high for a standard mixture of AR and its metabolites in acetonitrile (A) and of 1A2N6S in water showing its evolution with time (B).

**Supplementary Figure S7**. Effect of autoclave sterilization prior to faecal fermentation on AR stability. LC-MS chromatograms of BFM supplemented with AR at high (blue lines) and ADI (red lines) doses without (dashed lines) and with (solid lines) autoclaving.

**Supplementary Figure S8.** LC-MS chromatograms for faecal culture samples from donor 6 without addition of AR (blue lines) and with addition of AR at ADI and high doses (green and red lines, respectively). The Y-axis has been maintained at the same scale as in Figure S5 for comparison purposes.

**Supplementary Figure S9**. Analysis of the cytotoxic effect of the cell-free faecal supernatants used in this study on the colonocyte HT29 cell line. The cell index (CI: impedance values) was continuously monitored using the xCellingence RTCA-DP equipment, along the formation of the cellular monolayers (from 0 h to 20 hours), and for more than 24 additional hours (from 20 h to 48 h) after the addition of 40% of the supernatants in McCoy’s medium (MM) or MM supplemented with the highest concentration tested for each additive (**A**). The normalized-CI was calculated by the RTCA-DP software for each well of the E-plates: at a given time point the CI was divided by the CI value obtained in the first measurement after the sample addition and finally all data were normalized to a base line corresponding (subtracting each normalized-CI from the normalized-CI of the reference MM sample); then, the normalized-CI of the control MM is the “0 line” represented by the pink-coloured line (**B**). Control media: MM (McCoy’s medium without additives), BFM (basal fermentation medium added to MM at 40%), BFM-MSG-ADI (monosodium glutamate 0.8 mM, added to MM at 40%), and BFM-AR-high (Allura Red 0.6 mM, added to MM at 40%). Cell-free faecal supernatants: in the basal samples (0 h) and control samples (24 h of incubation) no additives were added and in the remaining samples. In the cultured samples the doses of food additive added for faecal culturing were: 0.8 mM for MSG-ADI, 0.08 mM for MSG-low, 0.06 mM for AR-ADI and 0.6 mM for AR_high (**C**). Normalized-CI was calculated as describe in material and method section (**D**).

**Supplementary Table S1**. SCFAs determined by GC/FID in basal fermentation media inoculated with the faecal samples prior to inoculation (Basal), and following fermentation in the different fermentation media: Control (BFM without food additives); MSG-ADI (BFM with MSG at a dose equivalent to its ADI); MSG-low (BFM with MSG at a dose 10 times lower than its ADI); AR-ADI (BFM with AR at a dose equivalent to its ADI); and AR-high (BFM with AR at a dose ten-fold the ADI).

|  | **Fermentation groups**: Mean ± SD (**mM**) | | | | | |
| --- | --- | --- | --- | --- | --- | --- |
| **Metabolites** | **Basal** | **Control** | **MSG-ADI** | **MSG-low** | **AR-ADI** | **AR-high** |
| Acetic acid | 168.95±58.74 | 1392.16±352.37 | 1450.04±341.19 | 1473.27±338.12 | 1375.48±313.11 | 1346.59±256.78 |
| Propionic acid | 43.48±23.65 | 405.68±89.04 | 402.27±78.44 | 422.35±95.27 | 328.53±10.89 | 389.65 ±93.85 |
| Isobutyric acid | ND | 60.15±13.80 | 58.72±13.04 | 63.34±13.85 | 53.80±3.85 | 59.62±8.51 |
| Butyric acid | 21.64±13.95 | 560.13±62.16 | 550.36±35.82 | 553.03±31.53 | 480.35±95.49 | 505.83±107.74 |
| Isovaleric acid | 4.61±1.57 | 124.32±24.69 | 119.84±21.04 | 131.03±22.08 | 109.90±9.14 | 123.36±16.62 |
| Valeric acid | 0.29±0.05 | 161.16±99.99 | 160.26±95.33 | 175.64±102.41 | 156.40±108.42 | 131.24±111.50 |
| Caproic acid | ND | 91.76±97.57 | 99.99±107.07 | 104.83±100.74 | 93.62±97.98 | 91.30±94.33 |

ND: Non Detected

**Supplementary Table S2**. Metabolites derived from MSG and AR detected by HPLC/PDA and LC/MS, respectively in the faecal culture samples.

|  | **Fermentation groups**: Mean ± SD (**mM**) | | | | | |
| --- | --- | --- | --- | --- | --- | --- |
| **Metabolites** | **Basal** | **Control** | **MSG** | **MSG-low** | **AR-ADI** | **AR-high** |
| MSG | 0.913±0.122 | 0.008±0.002 | 0.005±0.002 | 0.005±0.002 | NA | NA |
| GABA | 0.020±0.0136 | 0.0048±0.0118 | 0.0042±0.010 | 0.0043±0.0106 | NA | NA |
|  |  |  |  |  |  |  |
| AR | ND | NA | NA | NA | ND | ND |
| 4A5M5M | ND | NA | NA | NA | 0.021±0.042 | 0.31±0.42 |
| 1A2N6S | ND | NA | NA | NA | ND | ND |

NA: non applicable; ND: non detected
